# Tumor compactness improves the preoperative volumetry-based prediction of the pathological complete response of rectal cancer after preoperative concurrent chemoradiotherapy

**DOI:** 10.18632/oncotarget.13855

**Published:** 2016-12-10

**Authors:** Che-Yu Hsu, Chun-Wei Wang, Chia-Chun Kuo, Yu-Hsuan Chen, Keng-Hsueh Lan, Ann-Lii Cheng, Sung-Hsin Kuo

**Affiliations:** ^1^ Division of Radiation Oncology, Department of Oncology, National Taiwan University Hospital Yun-Lin Branch, Yunlin, Taiwan; ^2^ Division of Radiation Oncology, Department of Oncology, National Taiwan University Hospital and National Taiwan University Cancer Center, Taipei, Taiwan; ^3^ Division of Radiation Oncology, Department of Oncology, Taiwan Medical University Hospital, Taipei, Taiwan; ^4^ National Taiwan University Cancer Center, National Taiwan University College of Medicine, Taipei, Taiwan; ^5^ Cancer Research Center, National Taiwan University College of Medicine, Taipei, Taiwan; ^6^ Graduate Institute of Oncology, National Taiwan University College of Medicine, Taipei, Taiwan

**Keywords:** rectal cancer, tumor compactness, pathologic complete remission, preoperative, chemoradiotherapy

## Abstract

In addition to clinical factors (tumor and node stage) and treatment factors (equivalent radiotherapy dose and chemotherapy regimen), we assessed whether different performances of various tumor volume measurements help predict the pathological complete response (pCR) of locally advanced rectal cancer (LARC) after preoperative concurrent chemoradiotherapy (CCRT). A total of 122 patients with LARC treated with a long course of CCRT, between December 2009 and March 2015, were enrolled in this bi-institutional study. Tumor delineation was based on standard T2-weighted magnetic resonance imaging or contrast-enhanced computed tomography before CCRT. Tumor compactness was defined as the ratio of the volume and the surface area. The tumor compactness-corrected TV (TCTV) was defined as the ratio of the real TV (RTV) and tumor compactness. Twenty-three (18.9%) patients had a pCR. Areas under the curve of the receiver operating characteristic for pCR prediction calculated using the RTV, cylindrical approximated TV (CATV), and TCTV were 0.724, 0.747, and 0.780, respectively. The prediction performance of TCTV was significantly more efficient than that of both RTV (*P* = 0.0057) and CATV (*P* = 0.0329). Multivariate logistic regression analysis revealed tumor compactness (*P* = 0.001), RTV (*P* = 0.042), and preoperative clinical nodal status (*P* = 0.044) as significant predictors of a pCR. In addition, poor tumor compactness was closely associated with lymphovascular space invasion (*P* = 0.008) and pathological nodal status (*P* = 0.003). For patients with LARC receiving preoperative CCRT, tumor compactness is a useful radiomic parameter for improving the volumetric based prediction model.

## INTRODUCTION

Preoperative concurrent chemoradiotherapy (CCRT) followed by total mesorectal excision is the standard treatment of locally advanced rectal cancer (LARC) [1]. Long-course radiation therapy (RT) administered for 5–6 weeks could cause tumor downstaging, including a pathological complete response (pCR) [1]. Patients achieving a pCR have longer disease-free survival than those without a pCR [2]. Therefore, a “watch and wait” approach instead of immediate surgery may be selected by certain patients with a favorable response, particularly those with a lower rectal tumor unsuitable for sphincter-preserving surgery [3]. At present, few robust predictors of a pCR exist because tumor response to neoadjuvant therapy is not always predictable and the biological mechanism underlying the response or resistance of rectal cancer to CCRT is not established.

One of the possible predictors of a pCR is the pre-CCRT tumor volume (TV), which has been evaluated in several studies [4–7]. The most precise method for TV evaluation is the measurement of the real TV (RTV) by contouring the lesion from every cross-sectional area of each tumor-containing slice through magnetic resonance imaging (MRI) or contrast-enhanced computed tomography (CT) and subsequently multiplying each cross-sectional area with the section thickness [4–6]. In addition, the tumor size and volume measured using the maximal tumor diameter alone or the maximal tumor diameter and length with the assumption of cylindrical geometry have been observed to be significantly associated with pCR after preoperative CCRT [7, 8].

**Figure 1:**
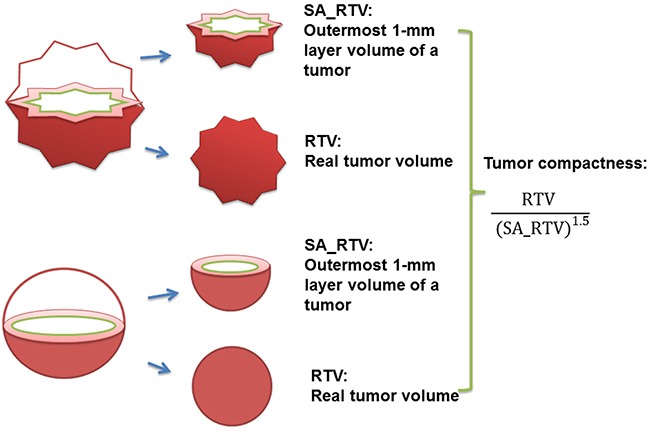
Illustration for RTV, SA_RTV, and tumor compactness (RTV, real tumor volume; SA_RTV, surface area of RTV)

Cylindrical approximated tumor volume (CATV), a crude measurement, is calculated from the maximum diameter and length, whereas the RTV, a direct measure of tumor burden, was measured slice by slice on MRI or CT examination. For example, the RTV of a spherical and a sea urchin-shaped tumor can be the same. However, in this example, CATV of the spherical tumor would clearly be smaller than that of the sea urchin-shaped tumor. The major difference between these two tumors is their compactness; tumor compactness is commonly defined as the ratio of the volume to the surface area (Figure 1). Aerts et al used noninvasive imaging techniques and demonstrated the correlation between various radiomic features, including tumor compactness, and the prognosis of lung as well as head and neck cancers [9]. Another recent study revealed that tumor compactness can differentiate triple-negative breast cancer from estrogen receptor-positive or Her2-positive breast cancer [10].

Noninvasive imaging techniques, such as tumor volume estimation, are potentially associated with CCRT response. Lambregts et al. reported that the areas under the curve (AUC) for the pCR prediction performance of preoperative and postoperative CCRT RTVs, defined by T2W-MRI in rectal cancer, were 0.77 and 0.82, respectively; they also reported that pre-CCRT MRI (AUC, 0.77) has a sensitivity of 55% and specificity of 74% in predicting pCR [6]. Appelt et al. incorporated radiation dose, CATV, and clinical nodal status in the prediction of treatment response for rectal cancer patients who received preoperative CCRT, and demonstrated that the preoperative CCRT CATV had a significant effect on the dose-response relationship for predicting tumor regression [7]. Nevertheless, in the volumetric based prediction model of rectal pCR, the correlation among tumor compactness, RTV, and CATV, and the role of tumor compactness in predicting the rectal pCR, remains uncertain. In this study, we sought to compare the pCR predictive performance of the RTV and CATV measurement methods for patients with LARC who received preoperative CCRT. We further analyzed whether volumetry-based tumor compactness is an independent predictor of a pCR in the same group of tumors.

## RESULTS

### Patient and treatment characteristics

We included 83 men and 39 women with a median age of 60.5 years (23-92.4 y). Considering the preoperative cT stage, 7 patients had cT2, 103 had cT3, and 12 had cT4 tumors. Furthermore, considering the preoperative cN stage, 31 patients had cN0, 55 had cN1, and 36 had cN2 diseases. According to the WHO classification of tumors of the digestive system, mucinous adenocarcinoma of colorectal carcinoma is defined as >50% of the tumor volume is composed of extracellular mucin. Tumors with a significant mucinous component >10% but <50% are usually termed adenocarcinoma with mucinous features or mucinous differentiation. In our cohort, 3 patients (2.5%) were diagnosed with mucinous adenocarcinoma, and 2 patients (1.6%) were diagnosed with adenocarcinoma with mucinous features. The tumors were located at 0–5, 5–10, 10–15 cm above the anal verge in 60, 57, and 5 patients, respectively (Table 1). A histopathological examination after preoperative CCRT revealed that 23 patients had ypT0, 12 had ypT1, 34 had ypT2, 51 had ypT3, and 2 had ypT4 tumors. Seventy-two patients showed primary tumor downstaging, and 61 showed nodal downstaging. Patients’ pathological features of tumors after preoperative CCRT are described in details in Supplementary Table 1.

**Table 1:** Baseline demographics of all patients

Demographics (N = 122)		Number	Percentage
Age	Median	60.5 years	23.0-92.4 years
Sex	Male	83	68 %
	Female	39	32 %
Clinical tumor stage (cT)	0	0	0
	1	0	0
	2	7	6 %
	3	103	84 %
	4	12	10 %
Clinical node stage (cN)	0	31	25.4%
	1	55	45.0%
	2	36	29.5%
Histology	Non-mucinous adenocarcinoma	117	95.9%
	Mucinous adenocarcinoma	3	2.5%
	Adenocarcinoma with mucinous feature	2	1.6%
Pre-OP CCRT to Surgery (interval)	3-6 weeks	29	24 %
	6-8 weeks	57	47 %
	>8 weeks	36	29 %
AAV	0-5 cm	60	49%
	5-10 cm	57	47%
	10-15 cm	5	4 %
CEA	Median	3.53	(0.3-102.5)
EQD2	<45	20	16.4%
	45-50	40	32.8%
	>50	62	50.8%
Chemotherapy	FL-based	112	92%
	Xeloda-based	10	8 %

Abbreviation: N, number; OP, operation; CCRT, chemoradiotherapy; EQD2, equivalent dose in 2 Gy fractions, AAV, above anal verge; FL, fluorouracil plus leucovorin

### Inter-observer variations analysis

The intra-class correlation coefficients of RTV between the original readers and reader 3 were 0.96 (MRI scan set) and 0.92 (CT scan set). The intra-class correlation coefficients of tumor compactness between the original readers and reader 3 were 0.81 (MRI scan set) and 0.84 (CT scan set). The intra-class correlation coefficients of CATV between the original readers and reader 3 were 0.68 (MRI scan set) and 0.73 (CT scan set). The intra-class correlation coefficients between RTV and tumor compactness were consistent (intra-class correlation coefficients > 0.75). The details of the inter-observer variability analysis are listed in Supplementary Table 2.

### RTV Volume, CATV, and tumor compactness

The median RTV and CATV were 27.25 cm^3^ and 71.14 cm^3^, respectively, whereas the median tumor compactness was 1.84 (Table 2). Table 2 shows the association between the RTV, CATV, and tumor compactness. Pearson and Spearman correlation analyses revealed no significant association between the RTV and tumor compactness. By contrast, the Pearson and Spearman correlation coefficients between the RTV and CATV were 0.806 (*P* < 0.0001) and 0.862 (*P* < 0.0001), respectively (Table 2).

**Table 2:** Characteristics and correlation analysis of tumor volumetry

Tumor volumetry characteristics	Median	Range
Long axis	5 cm	1.5-15 cm
Diameter	4.2 cm	2.02-8.17 cm
CATV	71.14 cm^3^	6.42-420 cm^3^
RTV	27.75 cm^3^	3.85-289.4 cm^3^
Tumor compactness	1.84	0.64-4.62
**Correlation analysis**	**Pearson method**	**Spearman method**
CATV vs. RTV	R = 0.806	R = 0.862
	*P* < 0.0001	*P* < 0.0001
CATV vs. Tumor compactness	R = -0.122	R = -0.217
	*P* = 0.141	*P* = 0.016
RTV vs. tumor compactness	R = -0.04	R = 0.129
	*P* = 0.633	*P* = 0.156

Abbreviation: CATV, cylindrical approximated tumor volume; RTV, real tumor volume

The Spearman correlation coefficient method revealed a significant association between the CAVT and tumor compactness (−0.217; *P* = 0.016). These results suggest a strong positive linear correlation between the RTV and CATV, a fairly negative nonlinear correlation between tumor compactness and the CATV, and an orthogonal correlation between the RTV and tumor compactness in patients with LARC receiving preoperative CCRT (Table 2).

### Predictors of pathological complete response after preoperative chemoradiotherapy

In univariate analysis, we found that positive clinical node (cN) was marginally associated with the pCR rate (node positive vs. negative, pCR 15.4% vs. 29.0%, *P* = 0.099), whereas clinical T stage (T4), EQD2 ≤ 50 Gy, and chemotherapy regimen were not associated with the pCR rate (Table 3). In contrast to clinical parameters, we discovered that among various tumor volume measurements, tumor compactness was significantly and positively correlated with rectal pCR (*P* = 0.001), whereas the RTV (*P* = 0.009) and CATV (*P* = 0.005) were significantly and negatively correlated with rectal pCR. Multivariate analyses revealed that tumor compactness (*P* = 0.001) was a good prognostic predictor of rectal pCR, whereas the cN status (*P* = 0.044) and RTV (*P* = 0.042) were negative predictors of rectal pCR (Table 3).

**Table 3:** Univariate and multivariate analysis for predictors of pCR after preoperative chemoradiotherapy

Pretreatment	Univariate analysis			Multivariate analysis		
Clinical factor	HR	*P* value	95% CI	HR	*P* value	95% CI
Age	0.982	0.301	0.949-1.016	0.967	0.160	0.922-1.013
Female	1.479	0.415	0.577-3.788	0.821	0.745	0.250-2.692
Pre-OP CCRT to surgery (interval)	0.907	0.746	0.502-1.639	1.009	0.979	0.497-2.050
cT stage 4 vs. 2 and 3	0.364	0.345	0.045-2.969	7.378	0.254	0.238-228.39
cN stage 1 and 2 vs. 0	2.252	0.099	0.860-5.889	3.701	0.044	1.033-13.259
EQD2 > 50 Gy vs ≤ 50 Gy	1.274	0.603	0.511-3.177	1.211	0.735	0.399-3.674
FL-based vs Xeloda-based	0.923	0.923	0.183-4.667	0.402	0.386	0.051-3.153
RTV	0.954	0.009	0.921-0.988	0.966	0.042	0.934-0.999
Compactness	3.368	0.001	1.672-6.786	4.103	0.001	1.801-9.346
CATV	0.981	0.005	0.967-0.994			

Abbreviation: HR, hazard ratio; CI, confidence interval; OP, operation; CCRT, concurrent chemoradiotherapy; CATV, cylindrical approximated tumor volume; RTV, real tumor volume; EQD2, equivalent dose in 2 Gy fractions; FL, fluorouracil plus leucovorin; Xeloda, capectiabine.

### Direct and model-assisted comparison of the pathological complete response predicted by using the RTV, CATV, and TCTV

On the basis of the aforementioned correlation analysis, the CATV could be considered a factor incorporating the RTV as a positive linear component, whereas tumor compactness acts as a negative nonlinear component. We further defined a new parameter called the tumor compactness-corrected TV (TCTV). The TCTV was defined as the ratio of the RTV and tumor compactness.

$$\text{TCTV}=\frac{\text{RTV}}{\text{Tumor compactness}}$$

**Figure 2:**
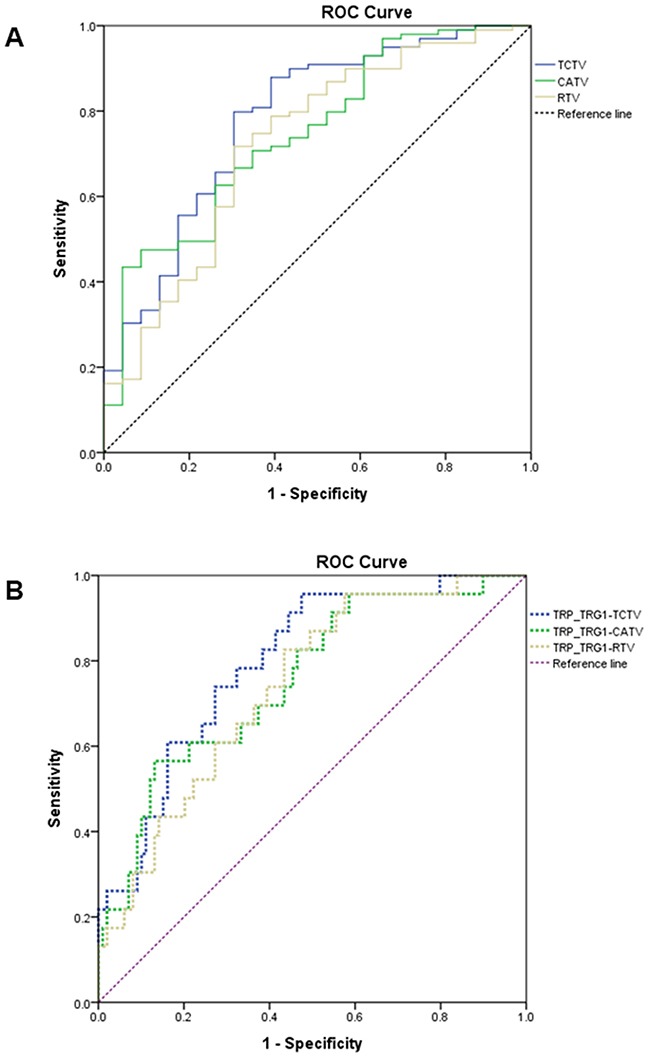
A. The blue, green, and yellow solid lines represent the ROC curve of the TCTV, CATV, and RTV-based prediction of the pCR **B**. The blue, green, and yellow dashed lines represent the ROC curve of the TRP–TRG ≦ 1–TCTV, TRP–TRG ≦ 1–CATV, and TRP–TRG ≦ 1–RTV prediction of the pCR (ROC, receiver operating characteristic; TCTV, tumor compactness-corrected tumor volume; CATV, cylindrical approximated tumor volume; TRP, tumor response probability; TRG, tumor regression grade; pCR, pathologic complete remission).

The receiver operating characteristic (ROC) curve analysis was performed to directly compare the pCR prediction performance by using different predefined TVs. The areas under the curves (AUCs) for pCR assessment by using the RTV, CATV, and TCTV were 0.724, 0.747, and 0.780, respectively. The ROC curve comparison revealed that the prediction performance of the TCTV was significantly more efficient than that of the RTV (*P* = 0.0057; Figure 2A and Table 4).

**Table 4:** Receiver operating characteristic curve analysis and comparison via pre-defined tumor volume

	AUC	95% CI
RTV	0.724	0.636 to 0.801
CATV	0.747	0.660 to 0.821
TCTV	0.780	0.696 to 0.850
**Pairwise comparison of ROC curves**		
RTV vs. CATV		
Difference between AUC	0.0224	
Significance level	*P* = 0.4735	
RTV vs. TCTV		
Difference between AUC	0.0553	
Significance level	*P* = 0.0057	
CATV vs. TCTV		
Difference between AUC	0.0329	
Significance level	*P* = 0.2751	

Abbreviation: AUC, areas under the curve; CI, confidence interval; CATV, cylindrical approximated tumor volume; RTV, real tumor; TCTV, tumor compactness-corrected tumor volume; ROC, receiver operating characteristic.

Appelt et al [7] developed a radiation dose–response model by incorporating clinical parameters, namely the radiation dose, CATV, and clinical nodal status: tumor response probability (TRP) and tumor regression grade (TRG):

$${\text{TRP}}_{T\text{RGf1}}=\frac{\exp(b0+b1EQD2+btumor-size*Yvol+bNstage*YN-stage}{1+\exp(b0+b1EQD2+btumor-size*Yvol\_bNstage*YN-stage}$$

TRP_TRG ≦ 1_ is the probability of a pCR. EQD2 is the equivalent dose of the tumor in 2 Gy per fractions. Y_vol_ is the preoperative CATV. The Y_N-category_ value is 0 for patients with clinical N0 and 1 for those with N1–2. Appelt et al also reported details on the b coefficients, namely b_0_, b_tumor size_,and b_N stage_, and other details of the model [7].

Because the Appelt et al. model incorporated radiation dose, CATV, and clinical nodal status in the prediction of treatment response for rectal cancer patients who received preoperative CCRT, we replaced CATV in the Appelt et al. model with RTV and TCTV. RTV and TCTV were normalized to have the same median volume as CATV to compare the prediction performance by using different predefined TVs. (Table 5). The AUCs for assessing the pCR using TRP_TRG ≦ 1_–CATV, TRP_TRG ≦ 1_–RTV, and TRP_TRG ≦ 1_–TCTV were 0.754, 0.738, and 0.795, respectively. The ROC curve comparison showed that the performance of TRP_TRG ≦ 1_–TCTV was significantly more favorable than that of both TRP_TRG ≦ 1_–RTV (*P* = 0.0029) and TRP_TRG ≦ 1_–CATV (*P* = 0.040; Figure 2B and Table 5).

**Table 5:** Receiver operating characteristic curve analysis and comparison via the assistance of model

	AUC	95% CI
TRP_TRG ≦1_-CATV	0.754	0.668 to 0.828
TRP_TRG ≦1_-RTV	0.738	0.650 to 0.813
TRP_TRG ≦1_-TCTV	0.795	0.713 to 0.863
**Pairwise comparison of ROC curves**		
TRP_TRG ≦1_-CATV vs. TRP_TRG ≦1_-RTV		
Difference between AUC		0.0162
Significance level		*P* = 0.4433
TRP_TRG ≦1_-CATV vs. TRP_TRG ≦1_-TCTV		
Difference between AUC		0.0413
Significance level		*P* = 0.040
TRP_TRG ≦1_-RTV vs. TRP_TRG ≦1_-TCTV		
Difference between AUC		0.0574
Significance level		*P* = 0.0029

Abbreviation: AUC, areas under the curve; CI, confidence interval; TRP, tumor response probability; TRG, tumor regression grade; CATV, cylindrical approximated tumorvolume; RTV, real tumor; TCTV, tumor compactness-corrected tumor volume; ROC, receiver operating characteristic.

### Subanalyses of ROC curves of RTV, CATV, TCTV for pCR prediction in two different subgroups, CT (n = 40) and MRI (n = 82)

We performed a subgroup analysis of ROC curves for the rectal pCR predictive power of RTV, CATV, and TCTV based on CT or MRI. The AUCs for MRI-based RTV, CATV, and TCTV in predicting rectal pCR were 0.732, 0.731, and 0.782, respectively. The TCTV prediction power was significantly better than RTV in the MRI only subgroup (*P* = 0.0061). The AUCs for CT-based RTV, CATV, and TCTV in predicting rectal pCR were 0.676, 0.828, and 0.770, respectively. There was a trend that TCTV prediction power was better than RTV in the CT only subgroup (*P* = 0.0773). These findings suggest that TCTV remains an important predictor for pCR in rectal cancer with preoperative CCRT irrespective of whether MRI or CT scans are used. All details of the subgroup analyses are shown in Supplementary Table 3 and 4.

### Subanalyses of ROC curves of RTV, CATV, TCTV for pCR prediction in non-mucinous adenocarcinoma subgroup

After excluding the 3 patients with mucinous adenocarcinoma (non-mucinous adenocarcinoma subgroup), we found that RTV, CATV, and tumor compactness were still significant predictors of a pCR in 119 patients in univariate analysis. In multivariate analysis, tumor compactness remained a significant predictor (*P* = 0.001), and RTV exhibited a trend (*P* = 0.051) to be a predictor of a pCR. The AUCs for RTV, CATV, and TCTV in predicting rectal pCR of the non-mucinous adenocarcinoma subgroups were 0.717, 0.740, and 0.774, respectively. The TCTV prediction power was significantly better than RTV for non-mucinous adenocarcinoma subgroup (*P =* 0.005; Supplementary Table 5 and 6).

### Predictors of other pathological features after preoperative chemoradiotherapy

Several studies have demonstrated that pathological nodal status (pN) and lymphovascular space invasion (LVSI) in post-CCRT pathologic specimens are closely associated with disease-free survival and overall survival in patients with LARC [11, 12]. Therefore, we assessed whether clinical parameters, including clinical stage and node, and treatment factors, including EQD2 and chemotherapy regimen, RTV, tumor compactness, and CATV were associated with pathological nodal status and LVSI in tumor specimens after preoperative CCRT. In univariate analysis, clinical nodal status was positively correlated to positive pathologic nodes (pN(+)) (*P =* 0.015) and tumor compactness was negatively correlated to pN(+) (*P* = 0.012) and closely associated with a less prominent LVSI (*P* = 0.004) (Supplementary Table 7 and Table 8).

Multivariate analysis revealed that the cN(+) status (*P* = 0.004) and tumor compactness (*P* = 0.003) remained significant predictors of pathological lymph nodes (LN) metastasis (Supplementary Table 7), and tumor compactness was only a predictor of less prominent LVSI (*P* = 0.008; Supplementary Table 8).

## DISCUSSION

Several studies have investigated the correlation between the preoperative TV and pCR of rectal cancer after preoperative CCRT [4–8]. According to a radiobiological principle, the tumor control probability is negatively correlated with number of cancer cells; therefore, theoretically, a large tumor size might be closely associated with a poor tumor response to RT [8]. In addition, large tumors might increase the heterogeneity of clonogenic cancer cells and aggravate their hypoxic condition, which are associated with an increased radioresistance [13–15]. The TV assessed on a slice-by-slice basis in cross-sectional MRI or CT is considered more reliable than that assessed using orthogonal tumor diameters and length. Lambregts et al [6] conducted a bi-institutional study and retrospectively determined the preoperative CCRT rectal TV on a slice-by-slice basis through T2-weighted MRI; they reported that the AUC of the ROC curve for pCR prediction was 0.73 (95% confidence interval: 0.61-0.86) [6]. Similar results using the measurement of rectal TV to validate the prognostic value of preoperative CCRT TV have been previously reported [4, 5, 16]. Nevertheless, Janjan et al [8] reported that the preoperative CCRT tumor size only determined using the maximum tumor diameter on the CT image also provided a reliable predictive value for tumor regression (*P* < 0.04), in which patients with a tumor size of <5 cm had >73% tumor regression rates. In addition, Appelt et al reported that the tumor size measured from the diameter and length on the preoperative MRI scan, assuming cylindrical geometry, was a crucial predictor of a pCR (*P* = 0.0399) [7].

The RTV encompassing the tumor burden slice by slice on MRI or CT is a more precise measure of the amount of space occupied by a rectal tumor, and we hypothesized that RTV may have a better prediction power than CATV, because CATV is a crude measurement based on maximum diameter and length. Our study compared the pCR prediction performance of the RTV and CATV, with the AUC of 0.724 and 0.747 (*P* = 0.475), respectively. The prediction performance of the preoperative RTV is in accordance with the result reported by Lambregts et al (AUC, 0.73) [6]. However, it remains unclear why the preoperative CATV, which is considered to be generated from a less precise measurement, yielded a comparable prediction performance. Our explanation is that the CATV might compromise 2 crucial and independent predictors, the TV and tumor compactness, that were associated with tumor response to preoperative CCRT. This hypothesis is supported by our current findings that revealed an orthogonal correlation between the RTV and tumor compactness, whereas the CATV had a positive linear correlation with the RTV but negative nonlinear correlation with tumor compactness. Furthermore, multivariate analysis revealed that the RTV and tumor compactness were significantly associated with the pCR. Based on the aforementioned results, we developed the TCTV and revealed that the AUC of the ROC curve for the pCR prediction performance of the TCTV was 0.780, which was significantly more reliable than that of the RTV (*P* = 0.0057) and CATV (*P* = 0.0329). To the best of our knowledge, TCTV, incorporating the two important factors tumor burden and tumor shape (tumor compactness), has not previously been defined. Our results show that TCTV has better prediction performance than traditional volumetric measurements such as RTV and CATV for a pCR of rectal cancer to preoperative CCRT.

To determine the differences between the pCR prediction performance of the predefined TV measurements and to decrease the effects of other predictors, such as the cN status and radiation dose, we selected the mathematical model constructed by Appelt et al [7] for further validation in the study patients. This model was derived from 2 prospective trials [17, 18] and comprises 3 parameters, namely the radiation dose, cN status, and CATV. Our results revealed that the AUC of the ROC curve for model-assisted prediction performance by using the TCTV was 0.795, which was significantly more reliable than that yielded by the RTV (0.738; *P* = 0.0029) and 0.754 of the CATV (*P* = 0.040). This result indicated that the TCTV (in addition to tumor compactness) has a more robust pCR prediction performance than do the RTV and CATV.

In addition to tumor volume measurements, diffusion-weighted MRI (DWI), 18F-fluorodeoxyglucose positron emission tomography (PET), and voxel-based quantitative imaging features provide a more precise, functional, and textural heterogeneity information for rectal cancer patients who received preoperative CCRT [19–22]. Lambregts et al. reported that the AUC for the pCR prediction performance of preoperative and postoperative CCRT TVs, defined by DWI in rectal cancer, was 0.77 and 0.82, respectively; they also reported that post-CCRT DWI volumetry (AUC, 0.92) has a sensitivity of 70% and a specificity of 98% in predicting pCR, respectively [6, 16]. Moreover, the performance of metabolic TV in predicting a pCR through PET has been previously reported [19, 20]. Recently, Nie et al. developed a quantitative model based on multi-parametric MRI features incorporating volume-average based and voxel-based textural heterogeneity analysis to predict pCR in patients with LARC who received preoperative CCRT [22]. In their studies, the AUC could be improved to 0.84 for pCR prediction through systemic analyses of multi-parametric MRI features, whereas the AUC for pCR prediction ranged from 0.54–0.73 based on conventional volume-averaged analysis [22]. These findings suggest that additional studies should be conducted incorporating the present TCTV into functional image-derived volumetry, such as DWI and PET scans, and studies of textural heterogeneity based volumetric analysis to predict the pCR of patients with LARC receiving CCRT followed by surgery should be conducted.

Tumor compactness is a radiomic factor and is often considered to be associated with tumor invasiveness and morphology [9, 21, 23], which are affected by stabilizing mechanical forces and 3-dimensional diffusion gradients [21]. Tumor compactness, in addition to tumor invasiveness, has been considered a prognostic factor [9, 23, 24]. In addition to pCR prediction, the present study reported that tumor compactness is a significant predictor of the LVSI and pN(+) after preoperative CCRT. However, the mechanisms underlying tumor compactness affecting the invasiveness and radioresistance of rectal tumors remains unclear. The possible reasons for the association between tumor compactness and its biological relevance are as follows: (1) The E-cadherin–*β*-catenin pathway could affect the compactness of the tumor surface and invasiveness of a tumor [25]. (2) The WNT–β-catenin pathway was observed to play a role in mediating radioresistance [26]. Besides, several studies incorporating genetic expression profiles into the radiologic phenotypes have demonstrated a close association between the radiological features and gene expression patterns in a variety of different types of cancer [9, 27, 28]. For example, Aerts et al. [9] compared four types of radiomic features (I, statistics energy; II, shape compactness; III, grey level nonuniformity; IV, grey level nonuniformity HLH) and gene-expression patterns in 89 cases of lung cancer, and found that radiomic features are significantly associated with the expression patterns of different genes. In particular, there was a strong association between intratumor heterogeneity features (III and IV radiomic features) and the expression patterns of the cell cycling pathways. However, further studies on the correlation between radiologic parameters, such as tumor compactness or TCTV, and genomic signatures in patients with LARC receiving preoperative CCRT are necessary. Considering that tumor compactness would be the phenotype resulting from a genotypic abnormality, such as the β-catenin-related signaling pathway, additional studies are warranted to investigate the association between radiomics and genomics signatures in patients with rectal cancer receiving preoperative CCRT followed by surgery.

Previous studies have demonstrated that the clinical nodal status of rectal cancer is an important factor for predicting a pathologic complete response (pCR) in patients with rectal cancer who have received neoadjuvant chemoradiotherapy [7, 29]. In the current study, we analyzed whether the clinical nodal status can alter the performance of tumor compactness in predicting pCR in the same group of tumors. In multivariate analyses (Table 3), we found that tumor volume and compactness were still predictive factors for pCR. In addition, clinical nodal status was closely associated with the pCR rate, consistent with the findings of previous studies [7, 29]. These findings indicate that tumor volume and compactness are consistently predictive factors of pCR for rectal cancer patients who want to receive preoperative CCRT and preserve anal function.

Our study had some limitations. First, MRI was conducted for 68% patients, and tumor delineation of the remaining 32% patients depended on the contrast-enhanced CT assisted with endoscopic findings or endoscopic ultrasound. Second, the bowel preparation before MRI and CT was inconsistent in patients, and the rectal distention level would cause a deviation in the TV and compactness measurement. Despite these limitations, the performance of the RTV and CATV for pCR prediction was comparable; these results are in accordance with those of previous MRI-based studies [6–8]. The bi-institutional study design would improve the generalizability of these results.

In conclusion, our findings revealed a reliable performance of the CATV as well as the RTV for predicting a pCR in patients with LARC after preoperative CCRT. Moreover, the performance of the developed TCTV model for pCR prediction was more robust than that of CATV and RTV. Tumor compactness could be a usefully radiomic factor for improving the volumetry-based prediction of a pCR in patients with LARC receiving preoperative CCRT. Further applications of tumor compactness in the existing pCR prediction model may facilitate the selection of complete responders during preoperative CCRT and accelerate the decision making process during a wait-and-sees situation. This approach requires further clarification to elucidate the association between radiological and biological signatures in patients with rectal cancer receiving radiotherapy.

## MATERIALS AND METHODS

### Patients

This study included patients with LARC who received a long course of neoadjuvant treatment at 2 medical centers between December 2009 and March 2015. The enrollment criteria included (1) histologically proven rectal adenocarcinoma, (2) long-course preoperative CCRT, (3) availability of MRI or contrast-enhanced CT before preoperative treatment, and (4) no metastatic diseases. A total of 122 consecutive patients enrolled from the medical centers were included in this analysis.

The study protocol was approved by the Research Ethical Committee of National Taiwan University Hospital (NTUH: 201605011RIND). The patients' medical data were anonymized prior to access and analysis. The institutional review board has waived the need for written informed consent from study subjects because all potentially patient-identifying information was removed prior to data analysis.

### Preoperative chemoradiotherapy and surgery

All patients were treated with intensity-modulated RT by using 6- and 10-MV photons. The targets were defined on the basis of the recommendations of the International Commission on Radiation Units and Measurements report no. 62 [30]. The gross TV of the tumor (GTV-T) and lymphadenopathy (GTV-N) were delineated using information from diagnostic MRI or CT and endoscopic ultrasound (EUS). The high-risk clinical target volume (CTV_H) included the GTV-T and GTV-N (if any). The low-risk clinical target volume (CTV_L) included the GTV-T and GTV-N (if any) as well as presacral, mesorectal, common iliac, internal iliac, and external iliac (only in cT4 disease) LN. In addition, 20 patients only received CTV_L irradiation, and one of them received the prescribed dose of 44 Gy in 22 fractions, whereas others received the radiation dose of 45 Gy in 25 fractions. Forty patients received an additional dose of 5.4 Gy in 3 fractions to the CTV_H after 45 Gy was administered to the CTV_L. Forty-eight patients concomitantly received 47.5 Gy to the GTV-T and 45 Gy to the CTV_L through a dose painting technique, followed by a GTV-T booster of 5.7 Gy in 3 fractions. The remaining 14 patients received 2 more fractions of 1.8 Gy per fraction dose to GTV-T after the total of 50.4 Gy was administered to the CTV_H area.

CCRT was administered to the patients. In total, 112 patients received 5-fluorouracil (FU)-based chemotherapy, including oxaliplatin. The most common regimen of 5-FU-based chemotherapy was an intravenous injection of 3 cycles of 5-FU (2000 mg/m2/d) for 36 hours and leucovorin (200 mg/m2/d) for 2 hours at the first, third, and fifth weeks of RT. The remaining 10 patients received capecitabine (1250 mg/m2) twice a day at the first, second, fourth, and fifth weeks of RT.

After preoperative CCRT, surgical resection was mostly performed approximately 6–12 weeks (median: 7 wk). In total, 98 (80%) patients underwent low abdominal resection, and 24 (20%) underwent anterior perineal resection because the tumors were close to the sphincter. The post-CCRT pathology of surgical specimens was carefully reviewed by 2 experienced pathologists at both institutions for evaluating the tumor response and pathological characteristics.

### Tumor volume and compactness measurement

MRI DICOM files of 82 patients and CT imaging DICOM files of 40 patients before preoperative CCRT were examined in this study. MRI was performed using a 1.5-T GE Signa scanner with a phased-array body coil. The imaging protocol included standard two-dimensional T2-weighted fast spin-echo sequences in three orthogonal directions (sagittal, coronal, and axial) with a 38-cm field of view, a 4-mm section thickness, and a 1-mm intersection gap. The repetition time and echo time were 3284 and 100 mini seconds, respectively. The echo train length was 21, and the bandwidth was 31.25–kHz. The number of signal averages was two. The acquisition voxel size was 0.78 × 1.14 × 5.00 mm. The number of slices was around 20–30. Bowel preparation agents or spasmolytics are not routinely given to our patients.

These images were evaluated using Pinnacle version 9.2 by 2 independent radio-oncologists with an experience of 4 and 5 years. To take into account inter-observer variations when measuring the tumor volume via MRI or CT scans, we invited a third reader (a radio-oncologist who has 8 years of experience with radiotherapy for the treatment of rectal cancer) to contour the tumor from 35 randomly chosen images obtained from MRI and CT scans. They were all blinded to the patient clinical data and pathology reports.

**Figure 3:**
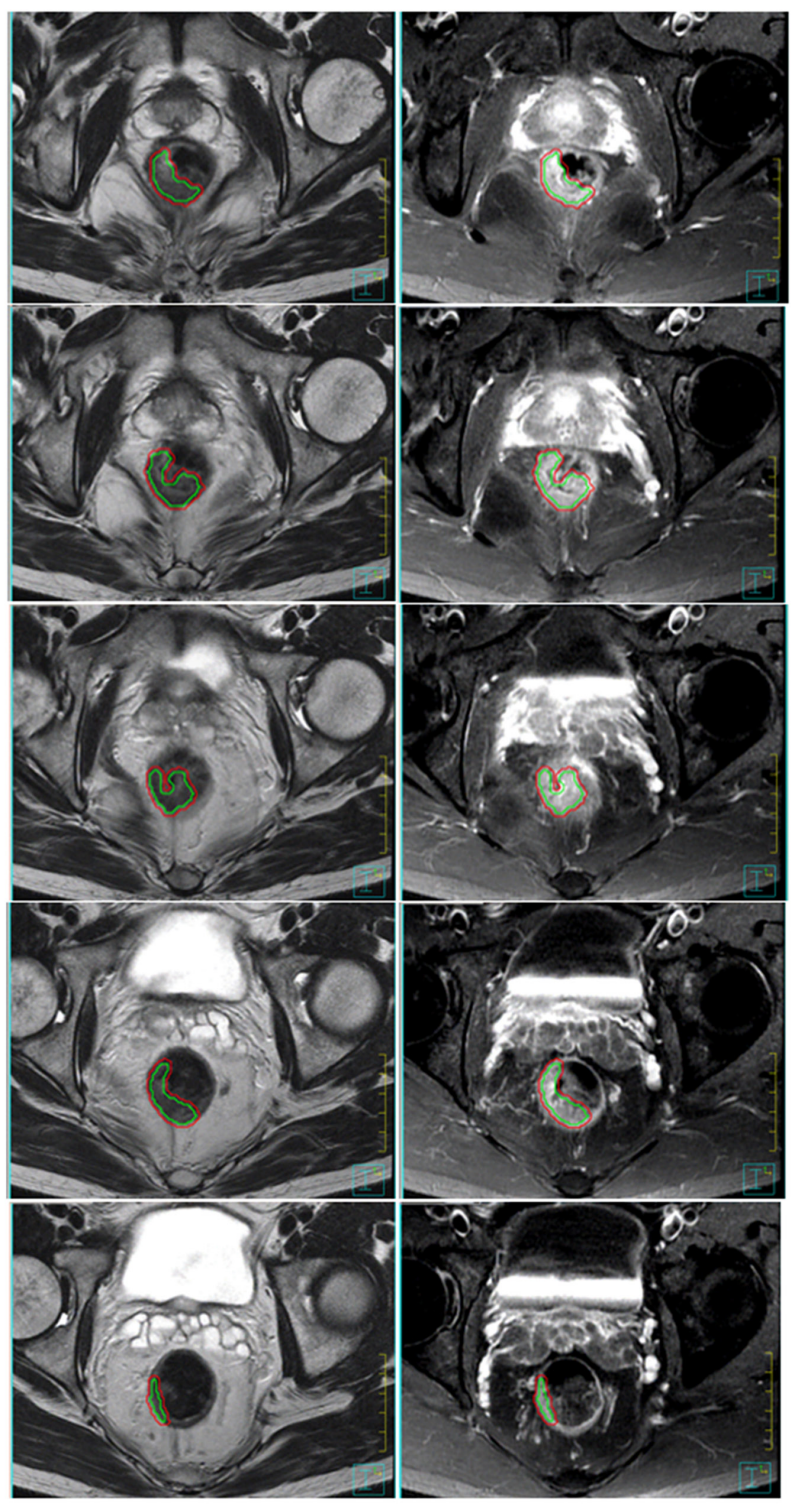
The rectal tumors of one patient are contoured as the ROI_RTV on the axial images (here, T2-weighted and contrast-enhanced T1-weighted MRI) for each tumor-containing lesion The red line encompasses the RTV area of every cross-sectional slice. The ROI_RTV_1 mm, the green line, was generated from the ROI_RTV with a 3-dimensional universal contraction of 1-mm length. The area between the outer red line and inner green line encompasses the outermost 1-mm layer volume as the surrogate of SA_RTV (RTV, real tumor volume; SA_RTV, surface area of RTV).

First, the rectal tumors were contoured as the region of interest (ROI), named the ROI_RTV (the red line in Figure 3) on the axial images for each tumor-containing slice. Second, we used the ROI contraction function of Pinnacle software to generate a new ROI called ROI_RTV_1 mm (the green line in Figure 3) from the ROI_RTV with a 3-dimensional universal contraction of 1-mm length. The definitions and the ratios of individual parameters are listed in Supplementary Table 9.

The RTV was estimated through the TV calculation function of the Pinnacle workstation to measure the volume within the ROI_RTV. The outermost 1-mm layer volume of a tumor could be derived from subtracting the volume within the ROI_RTV_1 mm from the volume within the ROI_RTV. We assumed the outermost 1-mm layer of the TV as the surrogate of the surface area of the RTV (SA_RTV). Thus, tumor compactness could be defined as $\frac{\text{RTV volume}}{{\left(\text{SA}\_\text{RTV}\right)}^{1.5}}$ [9, 10]. The maximum length and diameter of the tumor were also measured to calculate the CATV by assuming cylindrical geometry of tumors (Supplementary Table 9).

### Statistical analyses

Statistical analyses were performed using Statistical Package for Social Sciences (Version 21, Inc., Chicago, IL, USA) and MedCalc Version 11.2. Spearman correlation analysis was performed to determine the correlation between the RTV, the CATV, and tumor compactness. In the current study, we assessed the association between the pCR in patients who received preoperative CCRT and several potential predictors, including LVSI, pathological nodal status, age, sex, cT stage, cN stage, radiation dose, chemotherapy regimen, the interval period between CCRT completion and surgery, RTV, CATV, and tumor compactness (univariate analysis). However, in the model of multivariate logistic regression analysis, multicollinearity could exist when two or more of the predictors in a regression model are moderately or highly correlated. Considering that multicollinearity within the possible correlated predictors can lead to biased estimates and inflated standard errors, we checked multicollinearity among the aforementioned predictors before we performed the multivariate logistic regression analysis. We selected one of the common methods used for detecting multicollinearity via the Statistical Package for Social Sciences (Version 21, Inc., Chicago, IL, USA) to calculate the variance inflation factor (VIF) and to quantify how much the variance was inflated. As shown in Supplementary Table 10, the VIF of predictors from 2.5 to 10, including RTV (3.055) and CATV (3.470), were thought to be highly correlated with at least one of the other predictors in the aforementioned model. According to the results of Pearson and Spearman correlation analyses listed in Table 2, we found no significant association between RTV and tumor compactness. In contrast, the Pearson and Spearman correlation coefficients between RTV and CATV were 0.806 (*P* < 0.0001) and 0.862 (*P* < 0.0001), respectively (Table 2). Therefore, we chose RTV and compactness as predictors of further multivariate logistic regression analysis. When excluding CATV from the analysis of multicollinearity, we observed that the VIF of all predictors were less than 1.3 (Supplementary Table 11). In the study, we chose the predictors of age, sex, cT stage, cN stage, radiation dose, chemotherapy regimen, the interval period between CCRT completion and surgery, the RTV, and tumor compactness for further multivariate analysis.

ROC curves were generated to evaluate the prediction performance in detecting the pCR from a preoperative CCRT volume of different measurements and dose–response model developed by Appelt et al [7]. The ROC curve was compared using MedCalc Version 11.2 software based on a method described by Delong et al [31]. For the measurements performed by all readers (n=70), we evaluate the interobserver agreement via calculating the intraclass correlation coefficient (ICC), with ICC 0 to 0.39 indicating poor, 0.40 to 0.59 fair, 0.60 to 0.74 good, and 0.75 to 1.0 indicating excellent agreement [32].

### Abbreviation

pCR = pathological complete response, CCRT = concurrent chemoradiotherapy, LARC = locally advanced rectal cancer, RTV = real tumor volume, CATV = cylindrical approximated tumor volume, TCTV = tumor compactness-corrected tumor volume, AUC = areas under the curve, pre-CRT = Preoperative chemoradiation therapy, TME = total mesorectal excision, MR = magnetic resonance, CT = computed tomography, GTV-T = gross tumor volume of Tumor, GTV-N = gross tumor volume of lymphadenopathy, EUS = Endoscopic Ultrasound, CTV_L = low risk clinical target volume, CTV_H = high risk clinical target volume, 5-FU = fluorouracil, ROI = regions of interest, SA_RTV = surface area of RTV, LVSI = lymphovascular space invasion, ypN = pathological nodal status, ROC = Receiver operating characteristic, DWI = diffusion-weighted MR imaging, FDG = ^18^F-fluorodeoxyglucose, PET = positron emission tomography, TRP = tumor response probability, TRG = tumor regression grade (TRG)

## SUPPLEMENTARY MATERIALS FIGURES AND TABLES



## References

[1] Bosset JF, Collette L, Calais G, Mineur L, Maingon P, Radosevic-Jelic L, Daban A, Bardet E, Beny A, Ollier JC, Trial ERG (2006). Chemotherapy with preoperative radiotherapy in rectal cancer. N Engl J Med.

[2] Maas M, Nelemans PJ, Valentini V, Das P, Rodel C, Kuo LJ, Calvo FA, Garcia-Aguilar J, Glynne-Jones R, Haustermans K, Mohiuddin M, Pucciarelli S, Small W, Suarez J, Theodoropoulos G, Biondo S (2010). Long-term outcome in patients with a pathological complete response after chemoradiation for rectal cancer: a pooled analysis of individual patient data. Lancet Oncol.

[3] O'Neill BD, Brown G, Heald RJ, Cunningham D, Tait DM (2007). Non-operative treatment after neoadjuvant chemoradiotherapy for rectal cancer. Lancet Oncol.

[4] Kim YH, Kim DY, Kim TH, Jung KH, Chang HJ, Jeong SY, Sohn DK, Choi HS, Ahn JB, Kim DH, Lim SB, Lee JS, Park JG (2005). Usefulness of magnetic resonance volumetric evaluation in predicting response to preoperative concurrent chemoradiotherapy in patients with resectable rectal cancer. Int J Radiat Oncol Biol Phys.

[5] Yoon SM, Kim DY, Kim TH, Jung KH, Chang HJ, Koom WS, Lim SB, Choi HS, Jeong SY, Park JG (2007). Clinical parameters predicting pathologic tumor response after preoperative chemoradiotherapy for rectal cancer. Int J Radiat Oncol Biol Phys.

[6] Lambregts DM, Rao SX, Sassen S, Martens MH, Heijnen LA, Buijsen J, Sosef M, Beets GL, Vliegen RA, Beets-Tan RG MRI and Diffusion-Weighted MRI Volumetry for Identification of Complete Tumor Responders After Preoperative Chemoradiotherapy in Patients With Rectal Cancer: A Bi-institutional Validation Study. Ann Surg.

[7] Appelt AL, Ploen J, Vogelius IR, Bentzen SM, Jakobsen A (2013). Radiation dose-response model for locally advanced rectal cancer after preoperative chemoradiation therapy. Int J Radiat Oncol Biol Phys.

[8] Janjan NA, Crane C, Feig BW, Cleary K, Dubrow R, Curley S, Vauthey JN, Lynch P, Ellis LM, Wolff R, Lenzi R, Abbruzzese J, Pazdur R, Hoff PM, Allen P, Brown T (2001). Improved overall survival among responders to preoperative chemoradiation for locally advanced rectal cancer. Am J Clin Oncol.

[9] Aerts HJ, Velazquez ER, Leijenaar RT, Parmar C, Grossmann P, Carvalho S, Bussink J, Monshouwer R, Haibe-Kains B, Rietveld D, Hoebers F, Rietbergen MM, Leemans CR, Dekker A, Quackenbush J, Gillies RJ (2014). Decoding tumour phenotype by noninvasive imaging using a quantitative radiomics approach. Nat Commun.

[10] Agner SC, Rosen MA, Englander S, Tomaszewski JE, Feldman MD, Zhang P, Mies C, Schnall MD, Madabhushi A (2014). Computerized image analysis for identifying triple-negative breast cancers and differentiating them from other molecular subtypes of breast cancer on dynamic contrast-enhanced MR images: a feasibility study. Radiology.

[11] Kim NK, Kim YW, Min BS, Lee KY, Sohn SK, Cho CH Factors associated with local recurrence after neoadjuvant chemoradiation with total mesorectal excision for rectal cancer. World J Surg.

[12] Kim NK, Baik SH, Seong JS, Kim H, Roh JK, Lee KY, Sohn SK, Cho CH (2006). Oncologic outcomes after neoadjuvant chemoradiation followed by curative resection with tumor-specific mesorectal excision for fixed locally advanced rectal cancer: Impact of postirradiated pathologic downstaging on local recurrence and survival. Annals of surgery.

[13] Vaupel P (2004). Tumor microenvironmental physiology and its implications for radiation oncology. Semin Radiat Oncol.

[14] Hockel M, Schlenger K, Aral B, Mitze M, Schaffer U, Vaupel P (1996). Association between tumor hypoxia and malignant progression in advanced cancer of the uterine cervix. Cancer Res.

[15] Lim K, Chan P, Dinniwell R, Fyles A, Haider M, Cho YB, Jaffray D, Manchul L, Levin W, Hill RP, Milosevic M (2008). Cervical cancer regression measured using weekly magnetic resonance imaging during fractionated radiotherapy: radiobiologic modeling and correlation with tumor hypoxia. Int J Radiat Oncol Biol Phys.

[16] Curvo-Semedo L, Lambregts DM, Maas M, Thywissen T, Mehsen RT, Lammering G, Beets GL, Caseiro-Alves F, Beets-Tan RG (2011). Rectal cancer: assessment of complete response to preoperative combined radiation therapy with chemotherapy--conventional MR volumetry versus diffusion-weighted MR imaging. Radiology.

[17] Jakobsen A, Ploen J, Vuong T, Appelt A, Lindebjerg J, Rafaelsen SR (2012). Dose-effect relationship in chemoradiotherapy for locally advanced rectal cancer: a randomized trial comparing two radiation doses. Int J Radiat Oncol Biol Phys.

[18] Jakobsen A, Mortensen JP, Bisgaard C, Lindebjerg J, Hansen JW, Rafaelsen SR (2006). Preoperative chemoradiation of locally advanced T3 rectal cancer combined with an endorectal boost. Int J Radiat Oncol Biol Phys.

[19] van Stiphout RG, Valentini V, Buijsen J, Lammering G, Meldolesi E, van Soest J, Leccisotti L, Giordano A, Gambacorta MA, Dekker A, Lambin P (2014). Nomogram predicting response after chemoradiotherapy in rectal cancer using sequential PETCT imaging: a multicentric prospective study with external validation. Radiother Oncol.

[20] Schneider DA, Akhurst TJ, Ngan SY, Warrier SK, Michael M, Lynch AC, L Te Marvelde, Heriot AG (2016). Relative Value of Restaging MRI, CT, and FDG-PET Scan After Preoperative Chemoradiation for Rectal Cancer. Dis Colon Rectum.

[21] Frieboes HB, Zheng X, Sun CH, Tromberg B, Gatenby R, Cristini V (2006). An integrated computational/experimental model of tumor invasion. Cancer Res.

[22] Nie K, Shi L, Chen Q, Hu X, Jabbour SK, Yue N, Niu T, Sun X (2016). Rectal Cancer: Assessment of Neoadjuvant Chemoradiation Outcome based on Radiomics of Multiparametric MRI. Clinical cancer research.

[23] Leijenaar RT, Carvalho S, Hoebers FJ, Aerts HJ, van Elmpt WJ, Huang SH, Chan B, Waldron JN, O'Sullivan B, Lambin P (2015). External validation of a prognostic CT-based radiomic signature in oropharyngeal squamous cell carcinoma. Acta Oncologica.

[24] Martens MH, Subhani S, Heijnen LA, Lambregts DM, Buijsen J, Maas M, Riedl RG, Jeukens CR, Beets GL, Kluza E, Beets-Tan RG (2015). Can perfusion MRI predict response to preoperative treatment in rectal cancer?. Radiother Oncol.

[25] Ramis-Conde I, Drasdo D, Anderson AR, Chaplain MA (2008). Modeling the influence of the E-cadherin-beta-catenin pathway in cancer cell invasion: a multiscale approach. Biophys J.

[26] Woodward WA, Chen MS, Behbod F, Alfaro MP, Buchholz TA, Rosen JM (2007). WNT/beta-catenin mediates radiation resistance of mouse mammary progenitor cells. Proc Natl Acad Sci U S A.

[27] Kuo MD, Gollub J, Sirlin CB, Ooi C, Chen X (2007). Radiogenomic analysis to identify imaging phenotypes associated with drug response gene expression programs in hepatocellular carcinoma. Journal of vascular and interventional radiology JVIR.

[28] Lambin P, Rios-Velazquez E, Leijenaar R, Carvalho S, van Stiphout RG, Granton P, Zegers CM, Gillies R, Boellard R, Dekker A, Aerts HJ (2012). Radiomics: extracting more information from medical images using advanced feature analysis. Eur J Cancer.

[29] Garland ML, Vather R, Bunkley N, Pearse M, Bissett IP (2014). Clinical tumour size and nodal status predict pathologic complete response following neoadjuvant chemoradiotherapy for rectal cancer. Int J Colorectal Dis.

[30] (2010). Recording, Prescribing, and Reporting Photon-Beam Intensity-Modulated Radiation Therapy (IMRT): Contents. J ICRU.

[31] DeLong ER, DeLong DM, Clarke-Pearson DL (1988). Comparing the areas under two or more correlated receiver operating characteristic curves: a nonparametric approach. Biometrics.

[32] Cicchetti DV (1994). Guidelines, criteria, and rules of thumb for evaluating normed and standardized assessment instruments in psychology. Psychological Assessment.

