# Supplementary material for: Tumor compactness improves the preoperative volumetry-based prediction of the pathological complete response of rectal cancer after preoperative concurrent chemoradiotherapy
Source: Oncotarget. 2016 Dec 10;8(5):7921–34. doi: 10.18632/oncotarget.13855 (PMC5352371; doi:10.18632/oncotarget.13855)
Supplement: Supplementary file 1 [file oncotarget-08-7921-s001.pdf]

Tumor compactness improves the preoperative volumetry-based prediction of the pathological complete response of rectal cancer after preoperative concurrent chemoradiotherapy

SUPPLEMENTARY TABLES

Supplementary Table 1: Pathological features after preoperative chemoradiotherapy

Pathology (N = 122)		N	Percentage
Tumor response	pCR	23	18.9%
	Non-pCR	99	81.1%
Post-CCRT pathological tumour stage (ypT)	0	23	18.9%
	1	12	9.8%
	2	34	27.9%
	3	51	41.8%
	4	2	1.6%
Post-CCRT pathological nodal stage (ypN)	0	88	72.1%
	1	25	20.5%
	2	9	7.4%
T down staging		72	59.0%
N down staging		61	50%
CRM	Positive	6	4.9%
	Negative	116	95.1%
LVSI	Positive	24	19.7%
	Negative	98	80.3%
Perineural involvement	Positive	23	18.9%
	Negative	99	81.1%
Surgery	APR	24	19.7%
	LAR	98	80.3%

Abbreviation: N, number; pCR, pathologic complete remission; CCRT, concurrent chemoradiotherapy; CRM, circumferential resection margin; LVSI, lymphovascular space invasion; APR: abdominoperineal resection; LAR: lower anterior resection

Supplementary Table 2: Interobserver variability analysis

**Tumor volumetric Parameter measured in MRI only group (n=35)**	**Observer1/Observer 2**	**Observer 3**	**Intraclass Correlation Coefficient**
RTV (cm^3)	35.49±30.49	29.01±24.93	0.96
Tumor compactness	1.86±0.51	1.64±0.47	0.81
CATV (cm^3)	90.12±79.76	94.02±82.93	0.68
**Tumor volumetric Parameter measured in CT only group (n=35)**	**Observer1/Observer 2**	**Observer 3**	**Intraclass Correlation Coefficient**
RTV (cm^3)	43.95±31.01	44.04±34.07	0.92
Tumor compactness	2.06±0.81	2.08±0.85	0.84
CATV (cm^3)	110.67±61.09	115.01±88.63	0.73
**Tumor volumetric Parameter measured in MRI and CT group (n=70)**	**Observer1/Observer 2**	**Observer 3**	**Intraclass Correlation Coefficient**
RTV (cm^3)	39.72± 30.83	36.53±30.59	0.93
Tumor compactness	1.96±0.68	1.86±0.71	0.74
CATV (cm^3)	100.40±71.28	104.51±85.86	0.68

Abbreviation: CT, chemotherapy; RTV, real tumor volume; CATV, cylindrical approximated tumor volume

Supplementary Table 3: Receiver operating characteristic curve analysis and comparison via pre-defined tumor volume MRI only subgroup

**Tumor volumetric parameter measured in MRI subgroup (n=82)**	**AUC**	**95% CI**
RTV	0.732	0.623 to 0.824
CATV	0.731	0.622 to 0.823
TCTV	0.782	0.677 to 0.866
**Pairwise comparison of ROC curves**		
RTV vs. CATV		
Difference between AUC		0.000905
Significance level		$P = 0.978$
RTV vs. TCTV		
Difference between AUC		0.0498
Significance level		$P = 0.0061$
CATV vs. TCTV		
Difference between AUC		0.0507
Significance level		$P = 0.150$

Abbreviation: AUC, areas under the curve; CI, confidence interval; RTV, real tumor volume; CATV, cylindrical approximated tumor volume; TCTV, tumor compactness-corrected tumor volume; ROC, Receiver operating characteristic

Supplementary Table 4: Receiver operating characteristic curve analysis and comparison via pre-defined tumor volume in CT image subgroup

Tumor volumetric parameter measured in CT image subgroup (n=40)	AUC	95% CI
RTV	0.676	0.623 to 0.824
CATV	0.828	0.622 to 0.823
TCTV	0.770	0.677 to 0.866
**Pairwise comparison of ROC curves**		
RTV vs. CATV		
Difference between AUC		0.152
Significance level		*P* = 0.124
RTV vs. TCTV		
Difference between AUC		0.0931
Significance level		*P* = 0.0773
CATV vs. TCTV		
Difference between AUC		0.0588
Significance level		*P* = 0.604

Abbreviation: AUC, areas under the curve; CI, confidence interval; RTV, real tumor volume; CATV, cylindrical approximated tumor volume; TCTV, tumor compactness-corrected tumor volume; ROC, Receiver operating characteristic

Supplementary Table 5: Univariate and multivariate analysis of non-mucinous adenocarcinoma subgroup for predictors of pCR after preoperative chemoradiotherapy

Pretreatment	Univariate analysis			Multivariate analysis		
**Clinical factor**	**HR**	***P* value**	**95% CI**	**HR**	***P* value**	**95% CI**
Age	0.980	0.265	0.946-1.015	0.966	0.159	0.921-1.014
Female	1.485	0.411	0.578-3.817	0.813	0.734	0.247-2.676
Pre-OP CCRT to surgery (interval)	0.911	0.752	0.510-1.626	1.010	0.978	0.501-2.034
cT stage 4 vs. 2 and 3	0.391	0.383	0.047-3.219	7.417	0.250	0.244-225.05
cN stage 1 and 2 vs 0	0.462	0.117	0.177-1.212	3.652	0.046	1.024-13.024
EQD2 > 50 Gy vs ≤ 50 Gy	1.300	0.575	0.520-3.250	1.210	0.735	0.401-3.655
FL-based vs Xeloda-based	0.955	0.955	0.189-4.828	0.433	0.420	0.056-3.311
RTV	0.955	0.012	0.922-0.990	0.967	0.052	0.935-1.00
Compactness	3.387	0.001	1.670-6.870	4.092	0.001	1.804-9.285
CATV	0.981	0.006	0.968-0.995			

Abbreviation: HR, hazard ratio; CI, confidence interval; OP, operation; CCRT, concurrent chemoradiotherapy; CATV, cylindrical approximated tumor volume; RTV, real tumor volume; EQD2, equivalent dose in 2 Gy fractions; FL, fluorouracil plus leucovorin; Xeloda, capectiabine.

Supplementary Table 6: Receiver operating characteristic curve analysis and comparison via pre-defined tumor volume in non-mucinous adenocarcinoma subgroup

Tumor volumetric parameter measured in CT image subgroup (n=40)	AUC	95% CI
RTV	0.717	0.627 to 0.796
CATV	0.740	0.651 to 0.816
TCTV	0.774	0.689 to 0.846
**Pairwise comparison of ROC curves**		
RTV vs. CATV		
Difference between AUC		0.022
Significance level		*P* = 0.480
RTV vs. TCTV		
Difference between AUC		0.0575
Significance level		*P* = 0.0050
CATV vs. TCTV		
Difference between AUC		0.0349
Significance level		*P* = 0.254

Abbreviation: AUC, areas under the curve; CI, confidence interval; RTV, real tumor volume; CATV, cylindrical approximated tumor volume; TCTV, tumor compactness-corrected tumor volume; ROC, Receiver operating characteristic

Supplementary Table 7: Univariate and multivariate analysis for predictors of positive pathologic nodes after preoperative chemoradiotherapy

Pretreatment	Univariate analysis			Multivariate analysis		
**Clinical factor**	**HR**	***P* value**	**95% CI**	**HR**	***P* value**	**95% CI**
Age	0.999	0.965	0.970-1.030	1.022	0.265	0.983-1.063
Female	1.232	0.625	0.534-2.845	1.624	0.340	0.599-4.402
Pre-OP CCRT to surgery (interval)	0.780	0.475	0.396-1.536	0.520	0.237	0.176-1.537
cT stage 4 vs. 2 and 3	1.995	0.269	0.587-6.781	1.902	0.402	0.423-8.546
cN stage 1 and 2 vs. 0	4.822	0.015	1.358-17.122	10.081	0.004	2.057-49.396
EQD2 ≤ 50 Gy vs. > 50 Gy	1.495	0.325	0.671-3.330	1.956	0.188	0.720-5.311
FL-based vs. Xeloda-based	0.349	0.116	0.094-1.265	0.173	0.051	0.03-1.008
RTV	0.997	0.671	0.990-1.014	0.995	0.506	0.978-1.011
Compactness	0.387	0.012	0.090-0.625	0.252	0.003	0.102-0.623
CATV	1.001	0.807	0.996-1.006			

Abbreviation: HR, hazard ratio; CI, confidence interval; OP, operation; CCRT, concurrent chemoradiotherapy; CATV, cylindrical approximated tumor volume; RTV, real tumor volume; EQD2, equivalent dose in 2 Gy fractions; FL, fluorouracil plus leucovorin; Xeloda, capecitabine.

Supplementary Table 8: Univariate and multivariate analysis for predictors of lymphovascular space invasion after preoperative chemoradiotherapy

Pretreatment	Univariate analysis			Multivariate analysis		
**Clinical factor**	**HR**	***P* value**	**95% CI**	**HR**	***P* value**	**95% CI**
Age	1.008	0.660	0.974-1.043	1.001	0.961	0.961-1.042
Female	0.657	0.416	0.238-1.81	0.756	0.636	0.237-2.408
Pre-OP CCRT to surgery (interval)	1.209	0.340	0.819-1.784	1.173	0.457	0.770-1.788
cT stage 4 vs. 2 and 3	0.800	0.783	0.163-3.917	0.939	0.947	0.146-0.623
cN stage 1 and 2 vs. 0	0.613	0.323	0.233-1.616	0.738	0.597	0.239-2.276
EQD2 ≤ 50 Gy vs. > 50 Gy	2.000	0.210	0.677-5.909	0.921	0.874	0.332-2.551
FL-based vs. Xeloda-based	0.538	0.397	0.128-2.258	0.875	0.876	0.164-4.670
RTV	1.002	0.772	0.990-1.014	1.002	0.774	0.988-1.017
Compactness	0.237	0.004	0.090-0.625	0.259	0.008	0.096-0.699
CATV	1.001	0.807	0.996-1.006			

Abbreviation: HR, hazard ratio; CI, confidence interval; OP, operation; CCRT, concurrent chemoradiotherapy; CATV, cylindrical approximated tumor volume; RTV, real tumor volume; EQD2, equivalent dose in 2 Gy fractions; FL, fluorouracil plus leucovorin; Xeloda, capecitabine.

Supplementary Table 9: Summary of parameters definition and measurement method

Parameters	Definition and Measurement Method
Real tumor volume (RTV)	Contouring the lesion from every cross-sectional area of each tumor-containing slice through MRI or CT and subsequently multiplying each cross-sectional area with the section thickness
Cylindrical approximated tumor volume (CATV)	The maximum length and diameter of the rectal tumor are measured to calculate the CATV by assuming cylindrical geometry
Tumor compactness-corrected tumor volume (TCTV)	$\frac{\text{RTV volume}}{\text{Tumor compactness}}$
SA_RTV = surface area of RTV	Outermost 1-mm layer volume of a tumor
Tumor compactness	$\frac{\text{RTV volume}}{(\text{SA\_RTV})^{1.5}}$
$TRP_{TRG \leqq 1}$	Tumor response probability (TRP) of rectal pCR
Equation of $TRP_{TRG \leqq 1}$	$\frac{\exp(b0 + b1EQD2 + btumor-size*Yvol + bNstage*YN-stage}{1 + \exp(b0 + b1EQD2 + btumor-size*Yvol + bNstage*YN-stage}$
EQD2	Equivalent dose of the tumor in 2 Gy per fractions
Y-vol	Preoperative CATV.
YN-category	Clinical nodal status: cN0=0 & cN1,2=1

Supplementary Table 10: The variance inflation factors (VIFs) of the predictors in patients with locally advanced rectal cancer (LARC) who received preoperative CCRT

Predictors	VIF
Age	1.304
Female/Male	1.243
Pre-OP CCRT to surgery (interval)	1.096
N stage 1 and 2 vs. 0	1.181
cT stage 4 vs. 2 and 3	1.310
EQD2 > 50 Gy vs ≤ 50 Gy	1.150
FL-based vs Xeloda-based	1.128
Compactness	1.107
RTV	3.055
CATV	3.470

Abbreviation: HR, hazard ratio; CI, confidence interval; OP, operation; CCRT, concurrent chemoradiotherapy; CATV, cylindrical approximated tumor volume; RTV, real tumor volume; EQD2, equivalent dose in 2 Gy fractions; FL, fluorouracil plus eucovorin; Xeloda, capectiabine.

Supplementary Table 11: The variance inflation factors (VIFs) of the predictors in patients with LARC who received preoperative CCRT

Predictors	VIF
Age	1.294
Female/male	1.160
Pre-OP CCRT to surgery (interval)	1.095
cN stage 1 and 2 vs. 0	1.145
cT stage 4 vs. 2 and 3	1.210
EQD2 > 50 Gy vs ≤ 50 Gy	1.147
FL-based vs Xeloda-based	1.125
Compactness	1.069
RTV	1.220

Abbreviation: HR, hazard ratio; CI, confidence interval; OP, operation; CCRT, concurrent chemoradiotherapy; RTV, real tumor volume; EQD2, equivalent dose in 2 Gy fractions; FL, fluorouracil plus leucovorin ; Xeloda, capectiabine.
